# TCA precipitation and ethanol/HCl single-step purification evaluation: One-dimensional gel electrophoresis, bradford assays, spectrofluorometry and Raman spectroscopy data on HSA, Rnase, lysozyme - Mascots and Skyline data

**DOI:** 10.1016/j.dib.2018.01.095

**Published:** 2018-02-05

**Authors:** Balkis Eddhif, Nadia Guignard, Yann Batonneau, Jonathan Clarhaut, Sébastien Papot, Claude Geffroy-Rodier, Pauline Poinot

**Affiliations:** a Institut de Chimie des Milieux et des Matériaux de Poitiers (IC2MP), Université de Poitiers, CNRS, Equipe Eaux, Biomarqueurs, Contaminants Organiques, Milieux, (E-BiCOM), 4 rue Michel Brunet, TSA 51106, F-86073 Poitiers, France; b Institut de Chimie des Milieux et des Matériaux de Poitiers (IC2MP), Université de Poitiers, CNRS, Equipe Site Actif au Matériau Catalytique (SAMCat), 4 rue Michel Brunet, TSA 51106, F-86073 Poitiers, France; c Institut de Chimie des Milieux et des Matériaux de Poitiers (IC2MP), Université de Poitiers, CNRS, Equipe Synthèse Organique, 4 rue Michel Brunet, TSA 51106, F-86073 Poitiers, France; d CHU de Poitiers, 2 rue de la Miléterie, CS 90577, F-86021 Poitiers, France

## Abstract

The data presented here are related to the research paper entitled “Study of a Novel Agent for TCA Precipitated Proteins Washing - Comprehensive Insights into the Role of Ethanol/HCl on Molten Globule State by Multi-Spectroscopic Analyses” (Eddhif et al., submitted for publication) [1]. The suitability of ethanol/HCl for the washing of TCA-precipitated proteins was first investigated on standard solution of HSA, cellulase, ribonuclease and lysozyme. Recoveries were assessed by one-dimensional gel electrophoresis, Bradford assays and UPLC-HRMS. The mechanistic that triggers protein conformational changes at each purification stage was then investigated by Raman spectroscopy and spectrofluorometry. Finally, the efficiency of the method was evaluated on three different complex samples (mouse liver, river biofilm, loamy soil surface). Proteins profiling was assessed by gel electrophoresis and by UPLC-HRMS.

**Specifications Table**

**Table:** 

Subject area	*Chemistry*
More specific subject area	*Proteomics, protein purification, protein precipitation, trichloroacetic acid*
Type of data	*Tables, Figures*
How data was acquired	*Raman (LabRAM HR800UV confocal microspectrometer, Horiba Jobin Yvon, Kyoto, Japan)*
	*Bradford assay (DC Protein Assay, Biorad)*
	*Electrophoresis (ImageJ software)*
	*UPLC-HRMS (Accela LC pumps, Q-Exactive Hybrid Quadrupole-Orbitrap mass spectrometer equipped of an ESI source, Thermo Fisher Scientific, Waltham, MA, USA)*
	*MASCOT search engine (Matrix Science, London, UK; version 2.6.0) and Skyline software (MacCoss Lab, Washington, US; version 3.7.0.10940)*
	*ProteomeXchange Consortium with identifier PXD008110*
Data format	*Raw, analyzed and processed data*
Experimental factors	
Experimental features	*Proteins extraction was performed on 500* *mg of soil, 10* *mg of biofilm and 15* *mg of mouse liver as starting material according to protocols of Chourey et al.*[2]*, Huang et al.*[3]*and Song et al.*[4]*respectively.*
	*Proteins were precipitated with 25% (w/v) trichloroacetic acid (TCA).*
	*The washing of protein pellet was performed with three different agents (acetone, ethanol, or ethanol/HCl). The mixture was vortexed and kept at −20* °*C for 1* *h, centrifuged at 16,600* *g for 15* *min at 4* *°C. The resulting pellets were dried in a SpeedVac concentrator, solubilized in a 50* *mM of ammonium bicarbonate buffer containing 10* *mM of Tris. Proteins were subjected to trypsin digestion for 24* *h at 37* *°C. Digestion was stopped with formic acid before gel, bradford and mass analysis.*
Data source location	*Poitiers, France*
Data accessibility	*data are with this article*

**Value of the data**

• Data show a comprehensive evaluation of protein conformational changes throughout TCA precipitation and one single step purification with various solvents.
• Data highlight the efficiency of ethanol/HCl purification for TCA-precipitated proteins.

• Ethanol/HCl represents a quick and inexpensive purification agent for proteomics studies.
• Presence and variability of proteins are potential values to determine which purification method must be used for proteomics investigation.

## 1 Data

TCA precipitation is one of the most common and robust technique required for protein analyses [5], [6], [7]. However it leads to molten globule states which hamper the solubilization of proteins in aqueous buffers for mass spectrometry analysis.

### 1.1 Comparison of washing agents on standard solutions

**Fig. 1:**
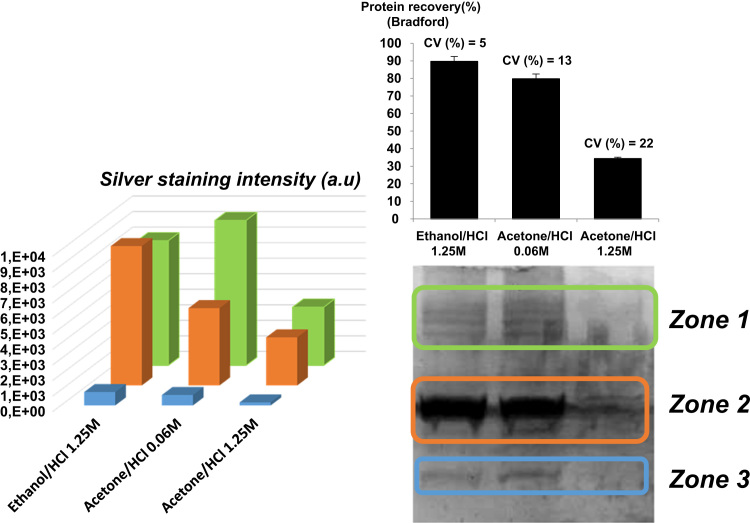
Standard proteins quantification by Bradford assay and silver-staining on electrophoresis gel. The thin line bars represent standard deviations at the top of the Bradford histogram. For both methods, histograms were constructed from the mean value of three independent assays.

A standard solution of HSA, cellulase (exoglucanases and endoglucanases mixture), lysozyme and ribonuclease A, 35 µg mL^−1^ each, was prepared in high purified water. Prot eins were precipitated with 25% (w/v) trichloroacetic acid (TCA) (final concentration). The clean-up of protein pellet was performed following three different approaches: ethanol/HCl (1.25 M; 3.8%), acetone/HCl (0.06 M; 0.2%); acetone/HCl (1.25 M; 3.8%) (Fig. 1).

### 1.2 Extraction and purification of endogenous proteins from complex sample matrices

See Fig. 2.

**Fig. 2:**
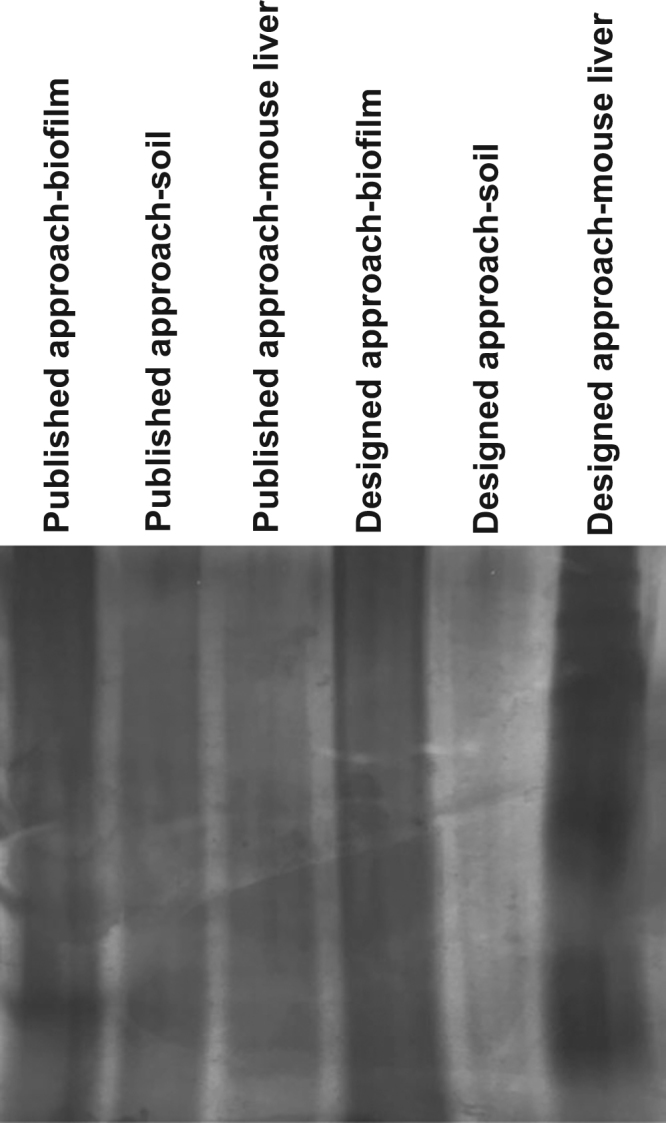
One-dimensional gel electrophoresis of complex matrices (biofilm, soil and mouse liver) after purification following the designed approach versus published protocols on complex matrices. The gel was stained with silver nitrate.

### 1.3 Effects of successive ethanol/HCl washings on proteins recoveries

10 mg of biofilm samples were spiked with the standard solution of HSA, exoglucanase 1 from the mix of cellulase, lysozyme, and ribonuclease A (Rnase). Proteins final concentration was 1 µg mg^−1^ of matrix to enable HRMS detection of the proteins after the whole process. The mixture was vortexed and left during 24 h at room temperature to favor proteins adsorption on the matrix. After extraction following the published protocol of Huang et al. [3], protein pellets were subjected to one, two or three ethanol/HCl washing(s).

They were then dissolved in 50 mM of ammonium bicarbonate containing 10 mM of Tris (pH 8.5), diluted in a ratio of 1:3 using the same buffer and subjected to trypsin digestion.

**Fig. 3:**
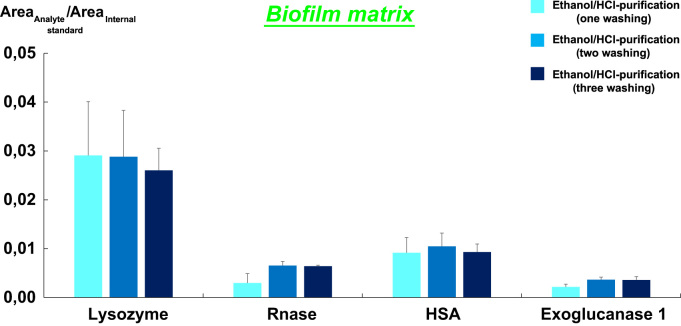
Proteins recoveries following the designed approach on biofilm sample. The thin line bars represent standard deviations at the top of each column. Each bar shows mean±s.e.m. from three independent purification assays. Protein recoveries in Tris buffer were determined by UPLC/HRMS in a full scan mode with a resolution of 70.000 and mass range of 200–3000 m/z.

Experiments were performed in triplicate. Fig. 3 gives the mean protein recoveries following the designed approach (Ethanol/HCl) on biofilm matrix after multiple washing steps.

### 1.4 Understanding the effect of ethanol/HCl on proteins conformation

#### 1.4.1 Spectrofluorometry

To get insights into the role of ethanol/HCl on proteins solubility, their conformational changes were comprehensively investigated, as an extension of the results reported in Ref. [1]. These measures were performed at each purification stage with two spectroscopic techniques: spectrofluorometry and Raman.

**Fig. 4:**
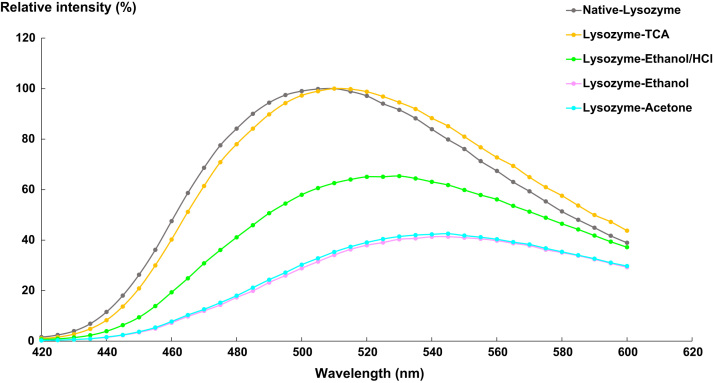
Emission spectra of lysozyme (λ_exc_ = 400 nm) at different purification steps. Native lysozyme (grey spectrum); Lysozyme-TCA (orange spectrum); Lysozyme-ethanol/HCl (green spectrum); Lysozyme-ethanol (purple spectrum); Lysozyme-acetone (blue spectrum).

**Fig. 5:**
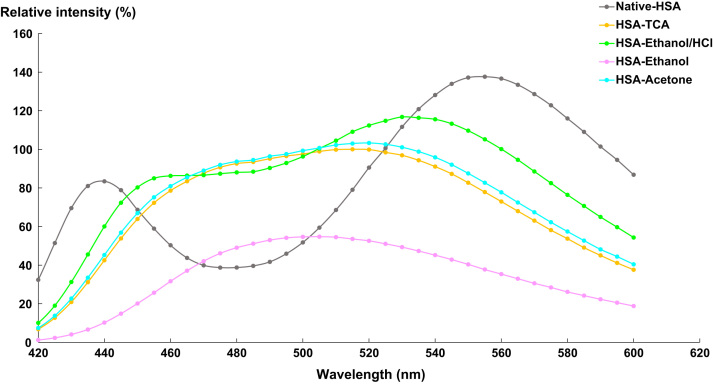
Emission spectra of HSA (λ_exc_ = 400 nm) at different purification steps. Native HSA (grey spectrum); HSA-TCA (orange spectrum); HSA-ethanol/HCl (green spectrum); HSA-ethanol (purple spectrum); HSA-acetone (blue spectrum).

**Fig. 6:**
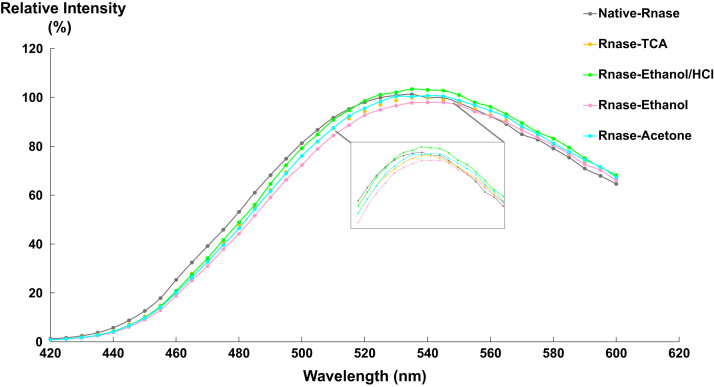
Emission spectra of RNASE (λ_exc_ = 400 nm) at different purification steps. Native Rnase (grey spectrum); Rnase-TCA (orange spectrum); Rnase-ethanol/HCl (green spectrum); Rnase-ethanol (purple spectrum); Rnase-acetone (blue spectrum).

Fig. 4, Fig. 5, Fig. 6 represent the fluorescence emission spectra of lysozyme, HSA and Rnase after TCA precipitation and washing steps (ethanol/HCl, ethanol or acetone).

#### 1.4.2 Raman microspectroscopy

**Fig. 7:**
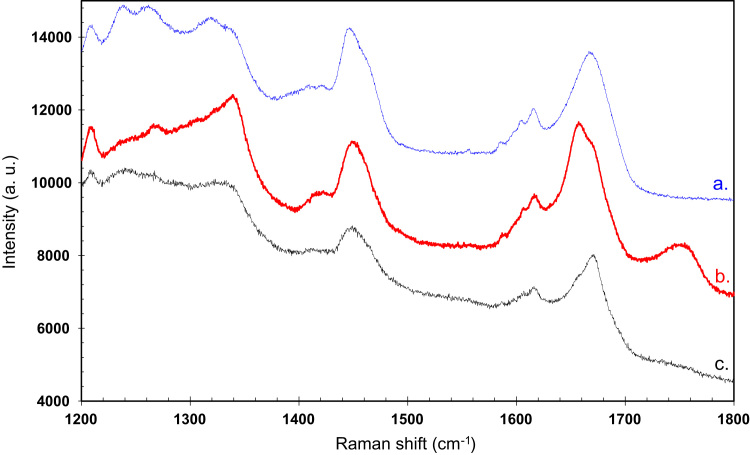
Raman spectra of Rnase at different purification steps (range 1200–1800 cm^−1^). a. Native Rnase (blue spectrum); b. Rnase-TCA (red spectrum) (shifted 1500 arbitrary units (a. u.) downward); c. Rnase-ethanol/HCl (black spectrum) (shifted 600 a. u. upward).

**Fig. 8:**
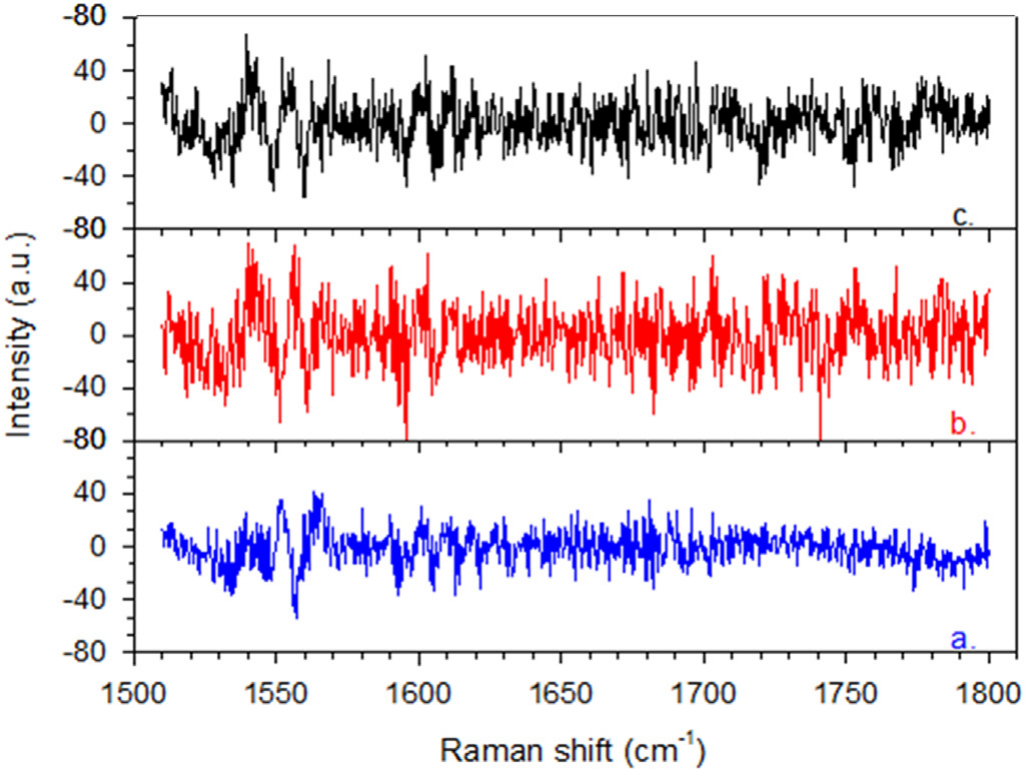
Difference spectra (experimental - fitting curve) after analysis of the amide I Raman bands of lysozyme at different purification steps (Fig. 5, [1]). a. Native lysozyme (blue); b. Lysozyme-TCA (red); c. Lysozyme-ethanol/HCl (black).

**Fig. 9:**
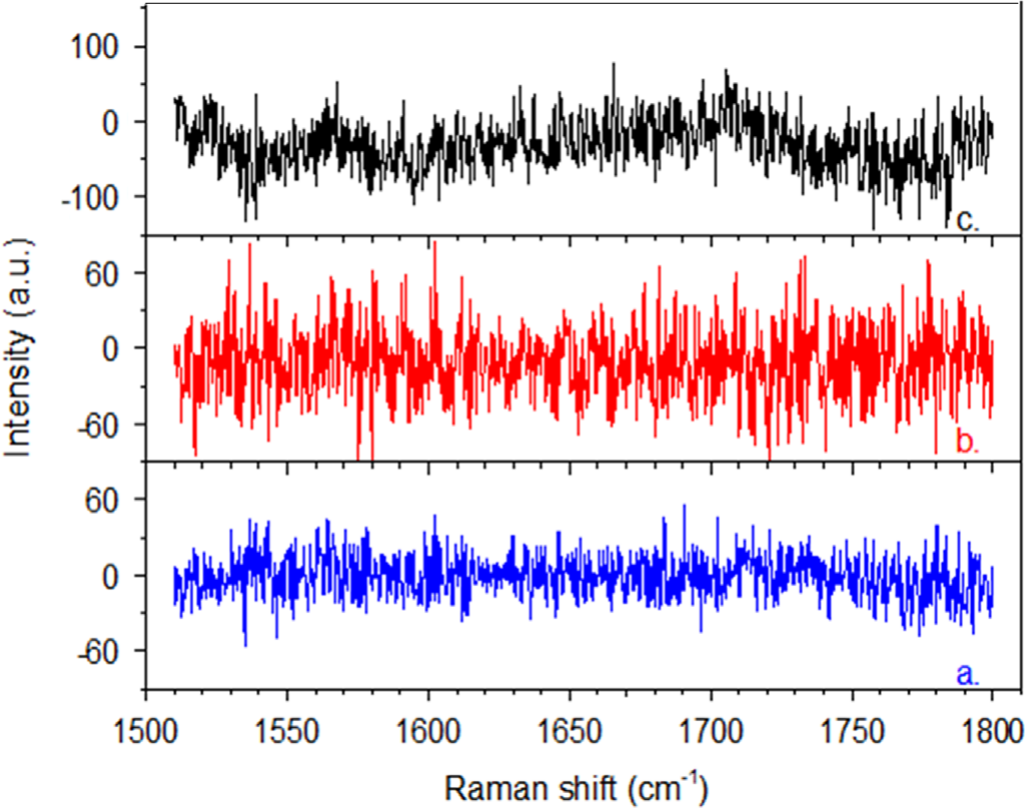
Difference spectra (experimental – fitting curve) after analysis of the amide I Raman bands of HSA at different purification steps (Fig. 6, [1]). a. Native HSA (blue); b. HSA-TCA (red); c. HSA-ethanol/HCl (black).

**Fig. 10:**
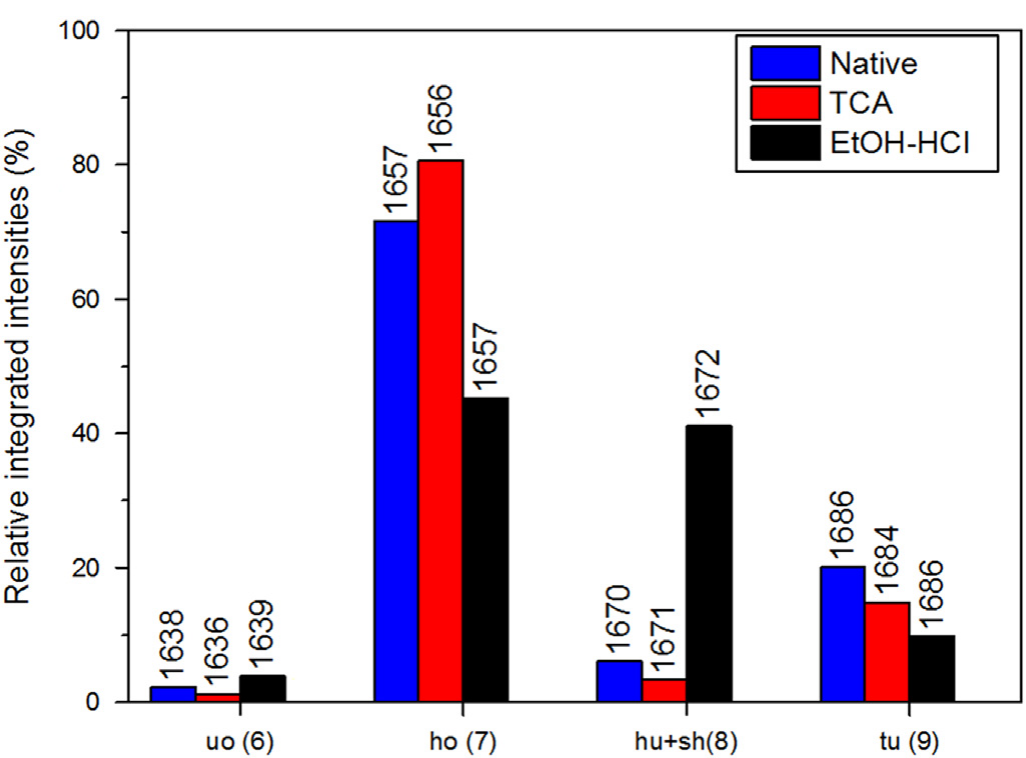
Relative integrated intensities of lysozyme amide I contribution from peak #6 assigned to unordered structures (*uo*), peak#7 (ordered α helices, *ho*), peak#8 (unordered α helices and β sheets, *hu*+*sh*), and peak #9 (turns, *tu*) as obtained after profile fitting of amide I region of the Raman spectra (Fig. 5, Ref. [1]). Values on top of each bar correspond to the Raman shift on which the contribution peak was centred at the end of the fitting.

**Fig. 11:**
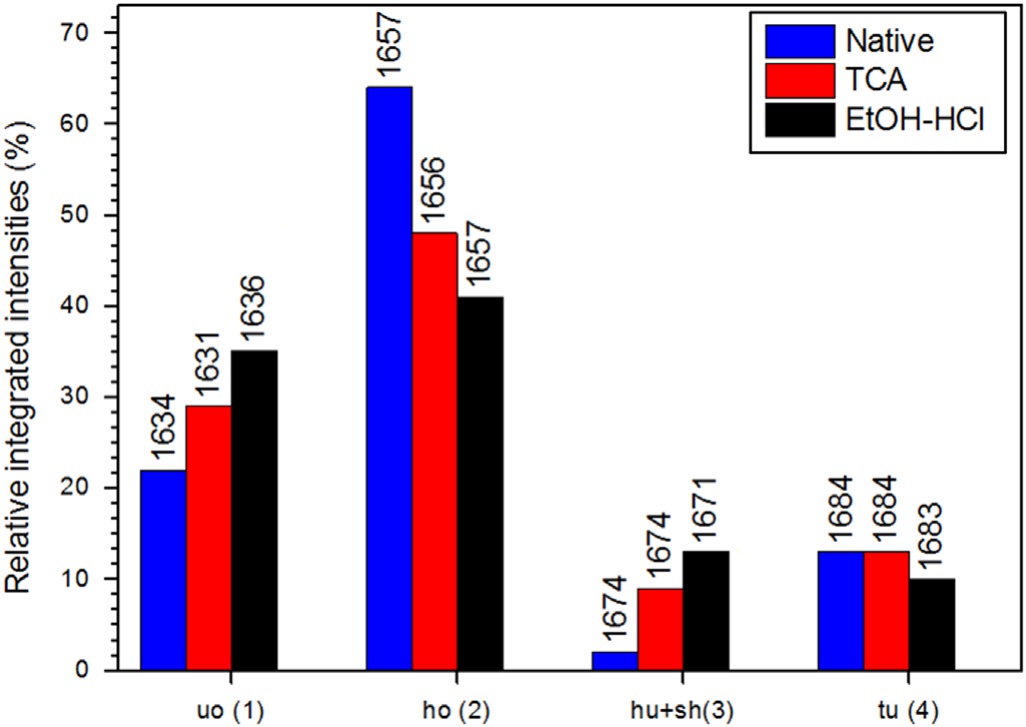
Relative integrated intensities of HSA amide I contribution from peak #1 assigned to unordered structures (*uo*), peak#2 (ordered α helices, *ho*), peak#3 (unordered α helices and β sheets, *hu*+*sh*), and peak #4 (turns, *tu*) as obtained after profile fitting of amide I region of the Raman spectra shown in Fig. 6[1]. Values on top of each bar correspond to the Raman shift on which the contribution peak was centred at the end of the fitting.

Raman spectrum for Rnase, is presented in Fig. 7. Spectra and curve fitting of the amide I band of proteins corresponding to lysozyme and HSA are presented in Fig. 5, Fig. 6 in Ref. [1], respectively (Fig. 8, Fig. 9, Fig. 10, Fig. 11).

The unfolding or aggregation of proteins usually involves some dynamic changes in their secondary structures. These changes are mainly monitored by the analysis of the amide I region (1600–1690 cm^−1^) which is assumed to be sensitive to α-helical secondary structures [8].

### 1.5 Extraction and purification of proteins from complex samples: LC-HRMS analysis

We present processed data of UPLC- HRMS analysis of proteins from different samples (mouse liver, river biofilm, soil) after TCA precipitation and solvent purification. The datasets in XML format can be used to evaluate ethanol/HCl purification for proteins profiling. Table 1 gives the HRMS features of peptides targeted for the standard proteins after in silico tryptic digestion. Table 2 presents endogenous proteins identified in soil, biofilm and mouse liver samples after purification following either the designed approach or published protocols (Mascot identification). Table 3 presents endogenous proteins detected in the mouse liver sample and quantified through Skyline with corresponding peptides and transitions for PRM. Table 4 presents endogenous proteins detected in the biofilm sample and quantified through Skyline with corresponding peptides and transitions for PRM (Table 5).

**Table 1:** HRMS features of peptides targeted for the four standard proteins after in silico tryptic digestion.

Protein name	Peptide sequence	[M+H]^1+^	[M+2H]^2+^	[M+3H]^3+^	[M+4H]^4+^
LYSO-1	FESNFNTQATNR		714.8288	476.8883	
LYSO-2	HGLDNYR	874.4166	437.7119	292.1437	
RNASE-1	CKPVNTFVHESLADVQAVCS QK			839.7457	630.0611
RNASE-2	HIIVACEGNPYVPVHFDASV		1112.5464	742.0334	
RNASE-3	YPNCAYK	915.4029	458.2051		
HSA-1	AVMDDFAAFVEK		671.8210	448.2164	
HSA-2	LVAASQAALGL	1013.5990	507.3031		
HSA-3	YLYEIAR	927.4934	464.2504	309.8360	
EXO-1	GSCSTSSGVPAQVESQSPNA K		1039.4764	693.3200	
EXO-2	YGTGYCDSQCPR		732.2876	488.5275	
EXO-3	VTFSNIK	808.4563	404.7282

**Table 2:** Endogenous proteins identified in soil, biofilm and mouse liver after purification following either the designed approach or the published protocols.

Sample	Location	Protein name	Phylogenetic origin	Protein coverage (%)		Score^a^		GRAVY	MW (Da)^b^
				The designed approach	Published protocol	The designed approach	Published protocol		
Soil	Extracellular region	Endoglucanase EG-II	*Hypocrea jecorina*	18	19	161	251	−0.19	44883
	Extracellular region	Xyloglucanase	*Hypocrea jecorina*	1	1	76	114	−0.21	87307
									
Biofilm	Cellular thylakoid membrane ; Peripheral membrane protein ; Cytoplasmic side	**C-phycoerythrin alpha chain**	***Microchaete diplosiphon***	**29**	**29**	**269**	**239**	−0.15	**17786**
	chloroplast thylakoid membrane ; Peripheral membrane protein By similarity; Stromal side	**R-phycoerythrin alpha chain**	***Porphyra purpurea***	**20**	**17**	**168**	**119**	−0.19	**17972**
	Cellular thylakoid membrane; Peripheral membrane protein ; Cytoplasmic side	**C-phycocyanin-1 alpha chain**	***Synechococcus sp,***	**17**	**17**	**181**	**177**	−0.11	**17335**
	Cellular thylakoid membrane ; Peripheral membrane protein ; Cytoplasmic side	**C-phycoerythrin alpha chain**	***Synechocystis sp,***	**20**	**20**	**209**	**176**	−0.12	**17756**
	Cellular thylakoid membrane ; Peripheral membrane protein ; Cytoplasmic side	Allophycocyanin alpha chain 1	*Microchaete diplosiphon*	11	11	76	84	−0.14	17411
	chloroplast thylakoid membrane ; Peripheral membrane protein ; Stromal side	B-phycoerythrin beta chain	*Porphyridium purpureum*	21	20	117	183	0.25	18884
	Cellular thylakoid membrane ; Peripheral membrane protein ; Cytoplasmic side	C-phycoerythrin beta chain	*Microchaete diplosiphon*	21	16	138	85	0.21	19568
	chloroplast thylakoid membrane ; Peripheral membrane protein ; Stromal side	R-phycoerythrin beta chain	*Pyropia haitanensis*	23	28	129	144	0.26	18810
	Cellular thylakoid membrane ; Peripheral membrane protein ; Cytoplasmic side	C-phycocyanin-1 beta chain	*Microchaete diplosiphon*	16	12	64	122	0.17	18080
	Cellular thylakoid membrane ; Peripheral membrane protein ; Cytoplasmic side	Allophycocyanin subunit alpha 1	*Nostoc sp,*	17	19	99	112	−0.09	17392
	chloroplast thylakoid membrane ; Peripheral membrane protein ; Stromal side	C-phycocyanin beta chain	*Aglaothamnion neglectum*	11	12	112	111	0.09	18290
	NI	Ribulose bisphosphate carboxylase large chain	*Trichodesmium erythraeum*	5	8	90	122	−0.32	53615
	Cellular thylakoid membrane ; Peripheral membrane protein ; Cytoplasmic side	Allophycocyanin alpha chain	*Anabaena cylindrica*	6	11	84	83	0.01	17128
	Cellular thylakoid membrane ; Peripheral membrane protein ; Cytoplasmic side	C-phycoerythrin alpha chain	*Pseudanabaena tenuis*	18	18	144	126	−0.24	17780
	chloroplast thylakoid membrane ; Multi-pass membrane protein	Photosystem II CP47 reaction center protein	*Odontella sinensis*	8	8	117	114	0.08	56436
	NI	Ribulose bisphosphate carboxylase large chain	*Cyanothece sp,*	9	6	94	89	−0.27	53531
	chloroplast	Ribulose bisphosphate carboxylase large chain (Fragment)	*Calyptrosphaera sphaeroidea*	5	9	90	107	−0.10	50919
	chloroplast	Ribulose bisphosphate carboxylase large chain	*Gracilaria tenuistipitata var, liui*	8	10	111	132	−0.10	54442
	chloroplast	Ribulose bisphosphate carboxylase large chain	*Cylindrotheca sp,*	6	6	109	108	−0.12	54400
	chloroplast thylakoid membrane ; Peripheral membrane protein ; Stromal side	Allophycocyanin beta chain	*Cyanidium caldarium*	13	16	94	83	−0.04	17574
	chloroplast	Ribulose bisphosphate carboxylase small chain	*Antithamnion sp,*	5	5	72	72	−0.58	16247
	NI	Carbon dioxide-concentrating mechanism protein CcmK homolog 1	*Synechocystis sp,*	18	29	71	72	−0.19	11128
	chloroplast thylakoid membrane ; Peripheral membrane protein ; Stromal side	R-phycoerythrin beta chain	*Aglaothamnion neglectum*	7	7	100	69	0.27	18710
	chloroplast	Ribulose bisphosphate carboxylase large chain (Fragment)	*Haptolina hirta*	9	10	141	139	−0.11	51098
	chloroplast	Ribulose bisphosphate carboxylase large chain	*Antithamnion sp,*	7	7	117	113	−0.12	54372
	Cellular thylakoid membrane ; Peripheral membrane protein ; Cytoplasmic side	Allophycocyanin beta chain	*Thermosynechococcus elongatus*	18	18	103	121	0.10	17462
	Cell inner membrane ; Multi-pass membrane protein	Photosystem I P700 chlorophyll a apoprotein A2	*Gloeobacter violaceus*	2	2	78	75	0.15	96126
	chloroplast thylakoid membrane; Peripheral membrane protein; Stromal side	Phycobiliprotein ApcE	*Aglaothamnion neglectum*	1	1	73	72	−0.23	101319
	NI	Ribulose bisphosphate carboxylase large chain	*Synechocystis sp,*	6	6	120	117	−0.29	53084
	chloroplast thylakoid membrane; Peripheral membrane protein; Stromal side	Allophycocyanin beta chain	*Galdieria sulphuraria*	16	16	96	73	0.02	17536
									
Mouse liver	Nucleus, Mitochondrion	**Carbamoyl-phosphate synthase**	***Mus musculus***	**39**	**33**	**1637**	**1268**	−0.12	**165711**
	Cytoplasm	**Arginase-1**	***Mus musculus***	**29**	**35**	**300**	**310**	−0.19	**34957**
	Cytosol, Nucleus,Membrane	**Selenium-binding protein**	***Mus musculus***	**31**	**28**	**526**	**405**	−0.31	**53147**
	Cytoplasm	**Argininosuccinate synthase**	***Mus musculus***	**32**	**15**	**429**	**191**	−0.11	**46840**
	Mitochondrion	**Glyceraldehyde-3-phosphate dehydrogenase**	***Mus musculus***	**31**	**32**	**321**	**298**	−0.04	**36072**
	cytosol	Cytosolic 10-formyltetrahydrofolate dehydrogenase	*Mus musculus*	9	17	139	361	−0.36	99502
	Extracellular region	3-ketoacyl-CoA thiolase, mitochondrial	*Mus musculus*	10	20	137	216	−0.38	42260
	Nucleus, Cytoskeleton,Cytosol	Serum albumin	*Mus musculus*	15	18	327	349	−0.09	70700
	Cytoplasm	Alcohol dehydrogenase 1	*Mus musculus*	19	29	161	212	0.20	40601
	membrane	Aspartate aminotransferase, mitochondrial	*Mus musculus*	15	16	231	215	−0.23	47780
	Endoplasmic reticulum	Carboxylesterase 3B	*Mus musculus*	12	14	201	183	−0.12	63712
	Cytoplasm	Glycine N-methyltransferase	*Mus musculus*	29	19	131	127	−0.25	33110
	membrane	Cytochrome P450 2D10	*Mus musculus*	9	2	100	123	−0.06	57539
	Cytoplasm	Aspartate aminotransferase, cytoplasmic	*Mus musculus*	7	13	112	115	−0.25	46504
	Cytoplasm	Adenosylhomocysteinase	*Mus musculus*	27	14	335	120	−0.07	47780
	Cytosol	Fructose-1,6-bisphosphatase 1	*Mus musculus*	12	16	117	120	−0.12	37288
	Endoplasmic reticulum	Carboxylesterase 3A	*Mus musculus*	13	9	220	139	−0.12	63677
	Mitochondrion	Sarcosine dehydrogenase, mitochondrial	*Mus musculus*	8	6	182	209	−0.25	102644
	membrane	UDP-glucuronosyltransferase 1-1	*Mus musculus*	4	8	94	141	0.09	60749
	Cytosol	Hemoglobin subunit beta-1	*Mus musculus*	16	24	111	105	0.08	15944
	Peroxisome	Peroxisomal bifunctional enzyme	*Mus musculus*	3	2	98	78	−0.12	78822
	membrane	Microsomal glutathione S-transferase	*Mus musculus*	17	21	80	87	0.15	17597
	membrane	Cytochrome P450 2F2	*Mus musculus*	6	7	128	130	−0.13	56141
	NI	Pyrethroid hydrolase Ces2a	*Mus musculus*	9	5	100	76	_	57539
	Extracellular region	Homogentisate 1,2-dioxygenase	*Mus musculus*	6	6	81	114	−0.34	50726
	Cytoplasm	Regucalcin	*Mus musculus*	4	13	72	112	−0.28	33899
	Peroxisome	3-ketoacyl-CoA thiolase B, peroxisomal	*Mus musculus*	13	8	116	84	0.05	44481
	membrane	Sorbitol dehydrogenase	*Mus musculus*	6	6	90	89	0.06	38795
	membrane	ATP synthase subunit f, mitochondrial	*Mus musculus*	26	26	70	71	−0.30	10394
	membrane	ATP synthase subunit alpha, mitochondrial	*Mus musculus*	14	10	193	160	−0.10	59830
	Cytosol	Urocanate hydratase	*Mus musculus*	2	1	100	76	−0.14	75227
	Extracellular region	Fumarylacetoacetase	*Mus musculus*	3	6	75	74	−0.21	46488
	Mitochondrion; Peroxisome	Uricase	*Mus musculus*	17	11	157	97	−0.46	35245
	Cytoskeleton	Fructose-bisphosphate aldolase B	*Mus musculus*	15	13	180	119	−0.26	39938
	membrane	UDP-glucuronosyltransferase 2B17	*Mus musculus*	11	6	104	96	−0.03	61386
	NI	Pyrethroid hydrolase	*Mus musculus*	9	7	108	89	−0.08	62356
	Cytoplasm	3-hydroxyanthranilate 3,4-dioxygenase	*Mus musculus*	9	6	90	87	−0.55	32955
	Mitochondrion	Hydroxymethylglutaryl-CoA synthase, mitochondrial	*Mus musculus*	7	6	86	70	−0.34	57300
	Mitochondrion	Trifunctional enzyme subunit alpha, mitochondrial	*Mus musculus*	9	7	90	81	−0.10	83302
	Endoplasmic reticulum	Microsomal triglyceride transfer protein large subunit	*Mus musculus*	1	1	74	80	−0.16	99664
	membrane	Cytochrome b-c1 complex subunit 2, mitochondrial	*Mus musculus*	4	4	73	76	−0.06	48262

a MASCOT score greater than 67.

b MW: Molecular weight.

**Table 3:** Endogenous peptides and transitions for PRM methods.

			PRM	
Protein name	Abreviattion	Peptide	Precursor(*m/z*)	Product (*m/z*)
Carbamoyl-phosphate synthase	CPSM	TAVDSGIALLTNFQVTK	898.4844	950.5306
				837.4465
				736.3988
		VLGTSVESIMATEDR	804.4009	1051.4725
				722.3138
				591.2733
		AFAMTNQILVER	696.8688	972.5473
				516.3140
				403.2300
		GQNQPVLNITNR	677.3653	926.5418
				617.3365
				390.2096
		AADTIGYPVMIR	653.8448	835.4495
				615.3647
				472.2402
		EPLFGISTGNIITGLAAGAK	644.0263	801.4829
				688.3988
				587.3511
		IALGIPLPEIK	582.3735	696.4291
				355.2340
				468.3180
		VMIGESIDEK	560.7814	890.4466
				777.3625
				231.1162
		SVGEVMAIGR	509.7711	832.4345
				646.3705
				547.3021
				
Argininosuccinate synthase	ASSY	EQGYDVIAYLANIGQK	891.4571	977.5415
				743.4410
				630.3570
		FELTCYSLAPQIK	785.4027	1085.4972
				556.3453
				485.3082
		QHGIPIPVTPK	593.8508	921.5768
				751.4713
				541.3344
		NQAPPGLYTK	544.7904	846.472
				775.4349
				314.1459
		YLLGTSLARPCIAR	530.9643	657.8692
				601.3271
				277.1547
				
Selenium-binding protein 2	SBP2	GSFVLLDGETFEVK	770.8983	1037.515
				924.4309
				809.404
		EEIVYLPCIYR	727.871	984.4971
				821.4338
				708.3498
		LTGQIFLGGSIVR	680.901	848.4989
				701.4304
				588.3464
		IYVVDVGSEPR	617.3273	957.5
				858.4316
				545.2678
		IFVWDWQR	575.2956	889.4315
				790.3631
				261.1598
		VIEASEIQAK	544.3033	875.4469
				746.4043
				675.3672
				
Glyceraldehyde-3-phosphate dehydrogenase	G3P	VPTPNVSVVDLTCR	778.9087	1259.6412
				949.4771
				630.3243
		WGEAGAEYVVESTGVFTTMEK	764.3561	912.4495
				892.4123
				756.3597
		GAAQNIIPASTGAAK	685.3753	815.4621
				702.3781
				668.3726
		LISWYDNEYGYSNR	593.9373	1021.4625
				539.2572
				376.1939
				
Arginase-1	ARGI1	VMEETFSYLLGR	722.8607	1214.6052
				855.4723
				708.4039
		EGLYITEEIYK	679.3479	1058.5405
				895.4771
				782.3931
		VSVVLGGDHSLAVGSISGHAR	673.3641	866.9581
				817.4239
				760.8819
		SLEIIGAPFSK	581.3293	606.3246
				556.3341
				478.266

**Table 4:** Endogenous peptides and transitions for PRM methods.

			PRM	
Protein name	Abreviattion	Peptide	Precursor(*m/z*)	Product (*m/z*)
R-phycoerythrin alpha chain, Porphyra purpurea	PHEA_PORPU	SVITTTISAADAAGR	717.3834	1134.5749
				1033.5273
				374.2146
		FPSSSDLESVQGNIQR	588.6235	715.3846
				621.2515
				587.3260
		NPGEAGDSQEK	566.2493	920.3956
				663.2944
				491.2460
				
C-phycocyanin-1 alpha chain, Synechococcus sp.	PHCA1_SYNP6	TPLTEAVAAADSQGR	743.8784	1175.5651
				945.4748
				775.3693
		FLSSTELQVAFGR	727.8855	1194.6113
				1107.5793
				790.457
				
C-phycoerythrin alpha chain, Synechocystis sp.	PHEA_SYNY1	TLGLPTAPYVEALSFAR	602.6647	1152.6048
				793.4203
				664.3777
		FPSTSDLESVQGSIQR	584.2917	688.3737
				635.2671
				560.3151
				
C-phycoerythrin alpha chain, Microchaete diplosiphon	PHEA_MICDP	SVVTTVIAAADAAGR	701.3834	1116.6008
				815.437
				374.2146
		ALGLPTAPYVEALSFAR	592.6612	1152.6048
				793.4203
				664.3777
		FPSTSDLESVQGSIQR	584.2917	688.3737
				635.2671
				560.3151

**Table 5:** Total spectrum, peptide and protein counts after purification by our approach versus published protocols on complex matrices.

	**Total spectrum count**	**Peptide count**	**Protein count**
Biofilm-published approach^a^	932	585	195
Biofilm-our approach^a^	937	424	163
Mouse liver-published approach^a^	1122	1408	416
Mouse liver-our approach^a^	959	1205	355
Soil-published approach^b^	946	293	72
Soil-our approach^b^	932	488	128

Data from the ProteomeXchange Consortium via the PRIDE [10] repository with the dataset identifier PXD0081110 and 10.6019/PXD008110.

a Average of three replicates.

b Counts of a single replicate.

## 2 Experimental design, materials and methods

Experimental design and materials and methods have been reported previously [1].
